# The Impact of Clinical Experience in Advanced Practice Nursing Education—A Cross-Sectional Study of Norwegian Advanced Practice Nurses’ Perspectives

**DOI:** 10.3390/nursrep13030110

**Published:** 2023-09-13

**Authors:** Ann-Chatrin Linqvist Leonardsen

**Affiliations:** 1 Faculty of Health, Welfare and Organization, Ostfold University College, P.O. Box (PB) 700, NO-1757 Halden, Norway; ann.c.leonardsen@hiof.no; 2 Faculty of Health and Social Sciences, University of Southeastern Norway, Raveien 215, 3184 Borre, Norway; 3 Department of Anesthesia, Ostfold Hospital Trust, P.O. Box 300, 1714 Grålum, Norway

**Keywords:** advanced practice nursing, continuing education, prerequisites

## Abstract

Background: An Advanced Practice Nurse (APN) is a specialized nurse who has acquired context specific knowledge, complex decision-making skills, and clinical competencies. Previously in Norway, APN education programs had a prerequisite of a minimum of two years of clinical nursing experience. Recently, the government decided to abandon this prerequisite. Objectives: The objectives of this study were to assess APN’s and APN students’ (1) perspectives on the impact of clinical nursing experience on various aspects of nursing practice, (2) perspectives on the association between APN students’ previous clinical experience and the ability to achieve the learning outcomes in the education program, and (3) attitudes towards clinical nursing practice as a prerequisite before entry to APN education programs. Design: An observational, cross-sectional design. Methods: APN and APN students were invited to respond to a researcher-developed questionnaire. A snowballing sampling method was used. The questionnaire included 24 questions scored on a five-point Likert scale, and two questions with text responses. Quantitative data were analyzed using descriptive statistics, and text responses with thematic analysis. Results: In total, 1767 APNs (92.9%) and APN students (7.1%) responded. Between 93.6 and 98.2% of the respondents (n = 1767) agreed that clinical nursing experience leads to experience with communication, collaboration, basic procedures, medical equipment and documentation, and to the development of situation awareness, increased awareness on own role in teams, the ability to provide person-centered nursing, an independent nursing identity, and feeling of security. Over 90% of the respondents agreed that students’ previous clinical experience was associated with the ability to achieve the learning outcomes in the APN program. In addition, 93.7% of the respondents agreed that clinical nursing experience should be a prerequisite before entry to APN programs. Advantages of clinical experience were reported as ‘Professional identity’, ‘Intuitive grasp’, ‘Integration of technical skills’, and ‘See the whole picture’. Disadvantages were reported as ‘Prejudice and bad habits’, ‘The importance of relevant experience’, and ‘Recruitment issues’. Conclusion: This study adds insights into the impact of clinical nursing experience as a prerequisite to APN education programs. The results indicate that clinical nursing experience is an essential contributor to the development of nursing competence and a nursing identity. This study was not registered.

## 1. Background

In March 2023, the International Council of Nurses (ICN) stated that the worldwide shortage of nurses should be treated as a global health emergency [1]. Among the suggestions to retain and recruit nurses were structured career opportunities, access to continuing education, implementing policies for improved career structures, and optimizing the workforce through advanced practice roles.

According to the ICN, an Advanced Practice Nurse (APN) is a generalist or specialized nurse who has acquired, through additional graduate education (minimum of a master’s degree), the expert knowledge base, complex decision-making skills, and clinical competencies shaped by central criteria and core competencies in a specific context [2]. The European Specialist Nurses Organisations’ (ESNO) vision is “to have qualified specialist nurses with academically accredited training programs to ensure safety and quality of care and mobility of workforce within Europe” [3]. This corresponds to a level 7 of education in the European Qualifications Framework (EQF) [4], or a masters’ degree level. However, different approaches and terms have been used to define APN roles, such as ‘nurse specialist’ or ‘clinical nurse specialist’, prepared in a clinical specialty at the master’s, post master’s, or doctoral level [4]. As such, the European Higher Education Area was established to facilitate student and staff mobility [5]. In total, 48 European countries, including Norway, have agreed to introduce a three-cycle higher education system consisting of bachelor’s, master’s, and doctoral studies. In Norway, an effort was initiated from 2019 to establish national guidelines in APN education programs to ensure a national standard, and that the courses cohere with international standards [6]. The government initiated a masters’ degree program of 120 European Credit Transfer System (ECTS) in advanced clinical general practice nursing [7], a brand new specialty in Norway. Traditionally, several nurse specialization programs existed, following regulatory attachments, or so called “framework plans” (in Norwegian: Rammeplaner). These courses varied from 60 to 90 ECTS. However, the government now claimed that these courses should comprise either 90 ECTS (as continuing education) or 120 ECTS (masters’ degree) at an APN level. The first guideline, in Public Health nursing, was implemented in July 2021 [8], followed by guidelines in critical care nursing [9], nurse anesthesia [10], operating room nursing [11], cancer nursing [12], and pediatric nursing [13]. The government also initiated a masters’ degree nursing program (120 ECTS) and a contining education program (90 ECTS) in mental health, intoxication, and addiction [14].

Prerequisites for entry to APN programs vary internationally [2,15,16,17]. Traditionally, a prerequisite before entry to the framework plan-guided APN courses in anesthesia, cancer, critical care, operating room, and pediatric nursing was ‘a minimum of two years relevant clinical nursing experience’. For the Public Health nursing program, the prerequisite was a minimum of one year. However, in the new national guidelines, this prerequisite was not included. This has led to worries in several of the professional environments, across clinicians and academics [18,19,20].

In 2011, Burns [21] identified a statistically significant positive relationship between prerequisites such as grades and critical care experience and academic progression for student registered nurse anesthetists. Chau et al. [22] found that active participation in clinical roles and knowledge translation was a crucial ingredient for promotion of nursing graduates to APN roles. Moreover, a 2023 scoping review [23] of literature exploring the impact of care experience prior to entry to pre-registration nurse education and training found that previous care experience had an impact on compassionate care, commitment, competence, and communication. However, the authors concluded that the evidence base was underdeveloped and suffered from several methodological limitations. Hence, no studies have explored the impact of clinical nursing experience as a prerequisite to APN educations beyond the study of Burns [21] on nurse anesthetists.

### Objectives

The objectives of the current study were to assess APN’s and APN students’ (1) perspectives on the impact of clinical nursing experience on various aspects of nursing practice, (2) perspectives on the association between students’ previous clinical experience and the ability to achieve the learning outcomes in the APN education programs, and (3) attitudes towards clinical practice as a prerequisite before entry to APN education programs.

## 2. Materials and Methods

### 2.1. Study Design

The study had an observational, cross-sectional design, using a researcher-developed questionnaire. The study adheres to the Strengthening the Reporting of Observational studies in Epidemiology (STROBE) checklist [24].

### 2.2. Setting and Context

The study was conducted throughout Norway, in various APN settings: anesthesia, cancer, critical care, operating room, and pediatric nursing environments. The study was conducted over eight weeks in July–August 2023.

### 2.3. Participants

A snowballing recruitment method was used [25]: Information about the study, including a link to a questionnaire, was published on the researchers’ FaceBook profile, and followers were asked to share this information extensively in their networks. Information about the study and a link to the questionnaire were also distributed through email in the researcher’s APN networks, with a request to share the email further. Eligibility criteria were APNs or APN students.

### 2.4. Data Sources

A questionnaire was developed based on studies focusing on nursing students and nurses’ technical skills and self-assessed competence [26,27,28,29], as well as supervisors of nurse anesthetist students’ perspectives on clinical nursing experience as a prerequisite to the APN education [18]. Based on this, statements regarding the impact of clinical nursing experience on various aspects of nursing practice (*n* = 15), perceived associations between APN students’ clinical nursing experience and the ability to achieve the learning outcomes in the APN education (*n* = 3), and about the necessity of clinical nursing experience before entry to an APN education program (*n* = 6) were developed. Response alternatives were on a five-point Likert scale, where 1 = totally disagree, 2 = disagree, 3 = neither disagree nor agree, 4 = agree, and 5 = totally agree. In addition, two text-alternative questions were added: ‘What is the advantage of having clinical experience previous to entry to an advanced nursing program?’ and ‘What is the disadvantage of having clinical experience previous to entry to an advanced nursing program?’.

Background variables collected were the type of specialization the respondent had or studied, and, to respondents experienced with supervising APN students, how many students they had supervised. See Supplement File S1 for the whole questionnaire.

### 2.5. Study Size

No sample size calculation was performed, since the aim was to include as many APNs and APN students as possible.

### 2.6. Statistical Methods

The Statistical Package for the Social Sciences (SPSS) version 28 was used to analyze the quantitative data. Descriptive statistics were used, and results are presented as n (%) or mean (standard deviation, SD). No methods for calculation of missing values were used, and the number of respondents is presented for each question.

A simplified thematic analysis, in-line with the recommendations of Braun and Clarke, was used to analyse the text responses [30]. First, all text responses were read in order to become familiarised with them (step 1). Then, the responses were inserted in a table with three rows (see Table 1) and coded line-by-line (step 2). The codes were then reviewed, searching for codes that were related and hence could be gathered in themes (step 3). Finally, the themes were reviewed and named (step 4).

### 2.7. Ethics

The study was based on the research ethical principles in the Declaration of Helsinki, on willing participation, informed consent to participate implicated by completion and submission of the questionnaire, anonymity, and confidentiality. According to Norwegian legislations, no approvals are necessary when data is anonymous, no sensitive information is collected, and patients are not included [31].

## 3. Results

### 3.1. Participants

In total, 1767 APNs/APN students responded to the questionnaire. Table 2 gives an overview of respondents’ characteristics.

Table 2 shows that the majority of the respondents were APNs (92%), and the majority of the respondents were either nurse anesthetists (37.4%) or operating room nurses (36.9). The majority currently worked as clinical nurses (71.8%).

### 3.2. Main Results

Table 3 gives an overview of responses to the question related to the impact of clinical nursing experience on various aspects of nursing practice.

Table 3 shows that between 93.6 and 98.2% of the respondents agreed that clinical nursing experience leads to experience with communication, collaboration, basic procedures, medical equipment and documentation, and to the development of situation awareness, increased awareness on own role in teams, of the ability to provide person-centered nursing, of an independent nursing identity, and feeling of security when providing nursing. Additionally, 78% agreed that clinical nursing experience leads to experience with advanced procedures.

Table 4 presents the respondents’ perception of the association between APN students’ previous clinical experience and their ability to achieve the learning outcomes in the APN education program. This question was directed to respondents with previous experience as a supervisor for APN students.

Table 4 shows that over 90% of the respondents perceived that students’ previous clinical experience was associated with the ability to achieve the learning outcomes in the APN education program. However, 12.4% agreed that students’ personal assets also had an impact.

Table 5 presents the respondents’ attitudes towards statements about clinical nursing practice as a prerequisite before entry to APN education programs.

Table 5 shows that 93.7% of the respondents agreed that clinical nursing experience should be a prerequisite before entry to APN education programs. This correlates with the fact that 88.9% disagreed that it should not be a prerequisite. Respondents also agreed that educational institutions should have similar prerequisites in order to ensure a national standard of APNs. However, regarding whether the clinical experience should be from specialized wards, the responses varied more: 71.3% of the respondents disagreed, were neutral, or agreed, respectively, on this issue.

### 3.3. Free-Text Responses

In total, 1193 respondents answered the question “What is the advantage of having clinical experience previous to entry to an advanced nursing program?” Through analysis, four themes were identified, namely ‘Professional identity’, ‘Intuitive grasp’, ‘Integration of technical skills’, and ‘See the whole picture’.

‘Professional identity’ was reported by many of the respondents as linked to a feeling of safety when providing treatment and care, both professionally and personally. One of the respondents wrote: ’One may not see own weaknesses and limitations before gaining more experience, and then look back on how unexperienced one was in different situations.’ Another respondent wrote: ‘Have seen patients in different situations, and learnt how oneself handles stressing and challenging situations’. Other statements were: ‘Build a minimum standard of comptence necessary to understand enough to handle new situations, and feeling safe doing it’, and ‘Experience with critically ill patients, clinical observation skills, safety in the nursing role, situational awareness, team experience, prepared for emergencies, know the equipment, lab results, digital journal systems, different drugs, CPR, emergency procedures and much more. This would be overwhelming for a new nurse and a barrier to acquire new and advanced knowledge and skills’.

‘Intuitive grasp’ was reported by some of the respondents as “clinical view” (in Norwegian: Klinisk blikk), something that needed years of experience to be developed. This was described by one of the respondents as: ‘You cannot read your way to a clinical view, and you don’t have enough time during the specialization to learn it. It makes it easier to focus on the essential in the education’. The words “Klinisk blikk” were used independently, and together with other terms, like “observations”, “experience”, “various patients”, or “various situations”. This again led to the fact that nurses knew what to do, based on previous experiences. In addition, this led to an ability to observe and assess the patients’ condition.

‘Intergration of technical skills’ was linked by some of the respondents to the development of competence in various technical skills, such as basic and advanced procedures, using various medical equipment, documentation, and drug administration. One of the respondents wrote: ‘Relevant practice will contribute to that the student doesn’t need to use time or energy learning basic procedures or acquire basic knowledge, but may focus on the specialization’. Another respondent wrote: ‘During the bachelor education one learns principles, theory and procedures, but it’s in clinical practice one gets volume training and experience. This is why clinical experience is essential, to be able to take the right decisions when facing critically ill patients, to ensure them safe and secure treatment’.

‘See the whole picture’ was related to all of the above themes, including both professional identity, intuition, and technical skills in the provision of person-centered nursing care. One respondent wrote: ‘Whole picture, associations, understanding’. The whole picture was related to both the patient pathway from prehospital, through the emergency department, via surgery to a ward, and then back to the municipality, and the whole patient, seeing individual needs, and being able to observe and assess the patients’ overall condition.

In total, 1173 respondents answered the question “What is the disadvantage of having clinical experience previous to entry to an advanced nursing program?” Of these, 548 responded “No disadvantages”. In addition, 13 respondents wrote ‘I don’t know’. Analysis of the remaining answers (n = 625) led to the identification of three themes, namely ‘Prejudice and bad habits’, ‘The importance of relevant experience’, ‘Recruitment issues’.

‘Prejudice and bad habits’ was reported as, for example, ‘Having meanings of their own, difficult to correct’, ‘Being colored by previous work culture’, ‘Established an inappropriate behaviour’, or ‘Think they know everything’. One of the respondents wrote: ‘It may happen that you’ve learnt something that must be unlearnt. The culture you take part in as a nurse matters. Different wards have different focus. I’ve experienced that some wards are sloppy with basic nursing…for example hygiene and the importance of proper nutrition in illness’. Another respondent wrote: ‘Experience may lead to prejudice, that they think they know the answer because they’ve seen something before’. These issues were reported as a disadvantage, comprising a barrier to new impressions, and nurses then being difficult to supervise.

‘The importance of relevant experience’ was linked to both that it could be difficult to get relevant clinical experience, and that the clinical experience nurses had before entry to the APN education program did not have any relevance to the specialization. Three of the respondents wrote: ‘It depends on what type of clinical experience one has’. Another respondent wrote: ‘If the clinical experience is irrelevant. One would have some experience from specialized wards, so one has seen very ill patients’. However, five of the respondents said that it could be difficult to get such experience because some specialized wards only recruited internally. One of the respondents also meant that the clinical nursing experience should be related to the specialization. The respondent exemplified this: ‘Clinical experience from a postoperative ward or an emergency department would in my opinion be more useful for a student nurse anesthetist, than experience from for example home-based services’. Irrelevant clinical experience was hence reported as a disadvantage leading to a lack of clinical awareness or lack of relatable experiences.

‘Recruitment issues’ was linked to both recruitment of APNs and recruitment to the APN education programs. Regarding recruitment of APNs, respondents perceived that requiring experience lead to ‘older nurses’, ‘nurses in the establishment phase, making it difficult economically to be a student’, or ‘nurses not used to study, having more difficulties with the academic level’. Moreover, a few of the respondents stated that requiring clinical experience would lead to longer educational paths for students. One of the respondents wrote: ‘It takes longer time to educate an APN’. Another respondent wrote: ‘It’s a disadvantage for the development of the profession, because we get educational courses so late in life…One may never get started, or start the education late in life’. Regarding recruitment to the education itself, some stated that nurses would choose not to specialize at all due to their age or economic situation. One of the respondents wrote: ‘Can be more difficult to recruit when other education programs allow going directly from a bachelors’ to a masters’ degree’.

## 4. Discussion

The majority of the respondents in the current study agreed that clinical nursing experience provides experience with various aspects of nursing, situational awareness, and an ability to work in teams. The majority also agreed that clinical nursing experience leads to the development of an individual nursing identity and a feeling of security in professional work performance. From the respondents’ perspective, this leads to an ability to provide person-centered nursing, increased efficacy, and patient safety. Most of the respondents also reported an association between APN students’ previous clinical experience and their ability to achieve the APN education program’s learning outcomes. In total, 93.7% of the respondents agreed that clinical nursing experience should be a prerequisite before entry to an APN education program in all educational institutions. The advantages of previous clinical experience were related to development of a professional identity, intuitive grasp, integration of technical skills, and an ability to see the whole picture. Disadvantages were related to the fact that students may have prejudice and bad habits, that their experience was not relevant to the speciality, and recruitment issues.

The majority of the respondents in the current study agreed that clinical nursing experience provides experience in various aspects of nursing practice, such as communication, medical equipment, basic and advanced procedures, documentation, and independent task solving. This is supported by previous research indicating that newly qualified nurses experience that the clinical demands are complex and overwhelming. They wish for higher competence in context-specific situations, and better knowledge about procedures they are expected to master [32,33,34]. Hence, such competence is assumed gained through clinical nursing practice after the end of the nurse bachelor program. Also, according to Benners’ model of skill acquisition, it takes newly qualified nurses at least five years to pass from advanced beginner level of proficiency to expert in nursing practice [35]. A 2022 systematic review [36] found that factors such as work experience, age, higher education, and work while studying affect the clinical competence of nurses. Hui et al. [37] found that clinical competency among nurses was moderate both before and during the COVID-19 pandemic. Before the pandemic outbreak, only shift type had an association with clinical competency. However, during the outbreak, work experience was associated with clinical competency. This also indicates that clinical nursing experience increases nurses’ ability to provide more effective and secure services, as indicated in the current study. If not requiring clinical nursing experience before entry to APN education programs, newly qualified nurses may enter directly after completion of the bachelor program. The consequences of such a change in a student’s professional foundation remains unexplored. However, respondents in the current study perceived that lacking clinical nursing experience would be associated with the APN students’ ability to achieve educational learning outcomes. This could again potentially impact the number of candidates completing the APN education, and hence the access to APNs.

Most respondents in this study agreed that clinical nursing experience develops situation awareness, team awareness, and an ability to provide person-centered nursing, which again develop an individual nursing identity and a feeling of security. Skills such as situational awareness and teamwork are central non-technical skills [38] that have been shown to increase both clinical efficacy and patient safety [39,40,41]. A 2023 study of APN students and supervisors within the emergency care, critical care, nurse anesthesia and operating room nursing specialties found that supervisors had higher focus on non-technical skills in clinical practice and on the impact on adverse events when compared to students [42]. This may indicate a link between clinical experience and the development of non-technical skills. In contrast, a 2020 systematic review [43] concluded that the level of evidence that postgraduate nursing qualifications improve outcomes for patients is weak. The authors suggest that future studies should move beyond examining nurses’ perceptions and should instead focus on empirical measures of the value of postgraduate education on nurse and patient outcomes.

The majority of the respondents in the current study agreed that clinical nursing experience should be a prerequisite before entry to APN education programs. This may be seen as obvious, bringing the responses to the remaining questions into the calculation. Of course, it may be assumed that APNs or APN students who are mainly positive to such a prerequisite chose to respond to the questionnaire, and hereby represent a selection bias. However, these findings add to the international knowledge base regarding prerequisites to APN education programs. Only two previous studies have been identified exploring similar aspects [21,22], both concluding that clinical experience is essential both for academic progression and for promoting nurses to APN roles. Hence, the current study underlines the importance of continued research exploring these issues. The majority of the respondents also agreed that Norwegian educational institutions should have similar prerequisites in order to ensure a national standard of APNs. This is in line with governmental intentions [6] and also with international intentions of similarities in higher education, allowing for an international workforce approach [44].

Several advantages and disadvantages with previous clinical nursing experience as a prerequisite for entry to APN education programs were identified. Advantages were related to development of a professional identity, intuitive grasp, integration of technical skills, and an ability to see the whole picture. These aspects align, to some degree, with the above mentioned study of competence and proficiency in nursing [35], non-technical skills development [45], and hereby development of an ability to ‘see the whole picture’. Disadvantages were related to the fact that students may have prejudice and bad habits, that their experience was not relevant to the speciality, and to recruitment issues. Previous studies have only explored prejudice in means of e.g., racism or ageism, and not as expressed by the respondents in the current study. As such, a 2020 review study [46] suggested that ‘talking about prejudice’ is implemented as part of educational programs for nurses. Requiring a minimum of two years of clinical nursing experience adds to the students’ age, and thereby the chance to have started a family, as suggested by respondents in the current study. Statistics in Norway [47] shows that the mean age of first-time parents is 30.2 years for females and 32.2 years for males, and hence would not necessarily be impacted through extending the path by two years. Moreover, respondents reported “longer educational paths” as a disadvantage. Also, Orsolini-Hain [48] claimed that prerequisites prolong the study pathway, leading to long waiting lists for further nursing education. To the authors’ knowledge, no other studies have been conducted presenting disadvantages of clinical nursing experience.

The ICN has underlined the importance of structured career opportunities, access to continuing education, and optimizing the workforce by APN roles, as contributors to the retention and attractiveness of nursing [1]. However, the lack of clinical skills and competencies have been shown to lead to high turnover rates, decreased job satisfaction, increased presenteeism, decreased patient safety, and increased medical errors [49,50]. Hence, it may be discussed whether it is appropriate to “shorten” the educational paths for APNs by not requiring clinical nursing experience before entry.

### Limitations

One of the major limitations of this study is that no validated questionnaire exists. However, the questionnaire was developed based on previous research findings by an APN professor well experienced with development and validation of questionnaires. A traditional, acknowledged, five-points Likert scale was used. Of course, the questionnaire could have been piloted to increase the face and content validity. Unfortunately, this was not done. As such, one of the respondents stated in text that it was ‘obvious what the questionnaire was intended to report, namely that clinical experience as a prerequisite before entry to an APN education program is necessary’. However, to avoid this, the questionnaire also included negatively loaded questions, as well as text alternatives. The same respondent claimed that nurses without a specialization also should have been invited to participate, since they may have important input.

Another major limitation is that the questionnaire was distributed through Facebook and emails. A 2017 systematic review exploring the use of Facebook as a recruitment method concludes that there is growing evidence to suggest that Facebook is a useful recruitment tool and its use, therefore, should be considered when implementing future health research [51]. Still, a selection bias may be present in the included sample. However, negative perceptions were expressed. It may also be argued that it is difficult for nurses without a specialization to assess the consequences of not having individual clinical nursing experience before entry to an APN education program or to APN practice.

The text responses were not very extensive, which limited a thorough analysis of the respondents’ perceptions and attitudes. Hence, a qualitative approach would have provided more in-depth information of APN and APN students’ perception of the impact of clinical experience, or lack thereof, before entry to an APN education program. The analysis was conducted by one researcher only, which limits the credibility and dependability of the analysis. However, a transparent description of the analytic steps, as well as the available dataset, will hopefully increase the trustworthiness of the results.

The snowballing recruitment method and the lack of sample size calculation may limit the generalizability of the study findings. Some of the specializations had very few respondents, and information about gender, age, or years of experience as an APN was not collected. A systematic review of national and other large-scale user experience surveys in local quality work found that the number of survey participants varied from 11 to 545 [52], which may indicate that the study sample is large enough to support the validity of the study findings.

The researcher is a nurse anesthetist with extensive clinical experience and experience as an educator and researcher. It may be claimed that this is a bias. However, the quantitative research design provided data that could be counted and analyzed statistically. The dataset is attached as Supplementary Material for others to explore, if desired.

## 5. Conclusions

The results of this study indicate that clinical nursing experience is an essential contributor to the development of professional competence and a nursing identity. APNs and APN students perceive that there is an association between previous clinical nursing experience and the ability to achieve APN education programs’ learning outcomes. Conclusively, the reasons for not including clinical nursing experience as a prerequisite for entry to APN programs should be carefully considered.

### Implications for Further Research

Even if the results in the current study are unambiguous, more research is needed, including information about respondents’ gender, age, and years of experience as an APN. The perspectives of APNs and APN students who do not support clinical nursing experience as a prerequisite should also be explored. Also, the perspectives of leaders and policymakers should be assessed. Further studies should also assess the outcomes of omitting clinical practice as a prerequisite to APN education, regarding e.g., whether students complete the programs, their competence directly after completion of the program, and also perhaps some years after the APN education.

## Figures and Tables

**Table 1:** Example of the analysis process of the text responses.

Responses	Codes	Themes
R1: Professional and personal safety, competence and experience with various patient groups, a trained clinical view, common professional language, better situation awareness, better collaboration across professions R2: Better clinical view, situation awareness, teamwork, communication with patients/colleagues, procedural understanding, care, get to know the profession, understand own responsibility, own security in the provision of care	Professional safety Personal safety Various experience Clinical view Professional language Situation awareness Collaboration Clinical view Situation awareness Teamwork Communication Procedures Know the profession Own responsibility Security	Professional identity Intuitive grasp The whole picture Integration of technical skills Professional identity

R = Respondent.

**Table 2:** Respondents’ characteristics (N = 1767).

APN/APN student, n (%) Student APN Missing, *n* = 9	125 (7.1) 1633 (92.9)
Specialization, n (%)	
Nurse anesthetist	660 (37.4)
Cancer nurse	228 (12.9)
Critical care nurse	122 (6.9)
Operating room nurse	652 (36.9)
Pedicatric nurse	13 (0.7)
No specialization	11 (0.6)
Emergency care nurse	10 (0.6)
Public health nurse	8 (0.5)
Midwife	4 (0.2)
General practitioner nurse	2 (0.1)
Other	68 (3.2)
Current position (N = 1596), n (%)	
Clinical nurse	1146 (71.8)
Teacher	52 (3.2)
Professional development	149 (9.3)
Leader	110 (6.9)
Researcher	17 (1)
Administration	32 (2)
Other	90 (5.8)
Respondents experienced with supervision of APN students (N = 1428)	
Number of students supervised, mean (SD)	11.9 (20.4)

APN = advanced practice nurse. SD = Standard deviation. Other specializations included geriatrics, management, mental health and addiction, neonatal, palliation, psycho-social, pulmonary, rehabilitation, scientific, and value-based nursing.

**Table 3:** Respondents’ perspectives on the impact of clinical nursing experience on various aspects of nursing practice (N = 1767). Clinical nursing practice leads to…

	Disagree n (%)	Neither Disagree nor Agree n (%)	Agree n (%)
Development of an individual nursing identity (missing, *n* = 9)	30 (1.7)	33 (1.9)	1695 (96.4)
Experience with communication with patients (missing, *n* = 8)	13 (0.7)	20 (1.1)	1726 (98.2)
Experience with communication with relatives (missing, *n* = 3)	11 (0.6)	52 (3)	1699 (96.4)
Experience with medical equipment (missing, *n* = 4)	27 (1.5)	76 (4.3)	1660 (94.2)
Experience with basic procedures (missing, *n* = 6)	19 (1)	37 (2.1)	1705 (96.9)
Experience with advanced procedures (missing, *n* = 8)	92 (5.2)	296 (16.8)	1371 (78)
Experience with interprofessional collaboration (missing, *n* = 9)	20 (1.1)	48 (2.7)	1690 (96.2)
Experience with documentation (missing, *n* = 7)	26 (1.5)	87 (4.9)	1647 (93.6)
Experience with independent task solving (missing, *n* = 10)	18 (1)	36 (2.1)	1703 (96.9)
Development of situational awareness (missing, *n* = 17)	17 (0.9)	34 (1.9)	1709 (97.2)
Development of awareness of own role in teams (missing, *n* = 9)	24 (1.4)	60 (3.4)	1675 (95.2)
Development of the ability to provide personcentred nursing (missing, *n* = 8)	15 (0.8)	98 (5.6)	1646 (93.6)
Increased patient safety (missing, *n* = 5)	20 (1.1)	55 (3.1)	1687 (95.9)
Increased efficacy (missing, *n* = 7)	31 (1.7)	135 (7.7)	1594 (90.6)
Development of a feeling of security in the provision of nursing care (missing, *n* = 6)	12 (0.7)	22 (1.2)	1727 (98.1)

Basic procedures = for example infusions, injections, drug administration, catheterization, venous access. Advanced procedures = for example continuous positive airway pressure support, non-invasive ventilation, suction of airways. Response alternatives Totally disagree (1) and Disagree (2) collated to ‘Disagree’. Response alternatives Agree (4) and Totally agree (5) collated to ‘Agree’.

**Table 4:** Respondents’ perspectives on the association between APN students’ previous clinical experience and the ability to achieve the learning outcomes in the APN educational program (N = 1767).

	Disagree n (%)	Neither Disagree nor Agree n (%)	Agree n (%)
There is no association between the number of years of clinical experience and students’ ability to achieve the learning outcomes (missing, *n* = 268)	1252 (83.5)	129 (8.6)	118 (7.9)
There is no association between the nature of the clinical experience and the students’ ability to achieve the learning outcomes (missing, *n* = 233)	1295 (84.4)	144 (9.4)	95 (6.2)
The students’ ability to achieve the learning outcomes depends more on personal assets than on clinical experience (missing, *n* = 215)	895 (57.7)	464 (29.9)	193 (12.4)

**Table 5:** Respondents’ attitudes towards various statements about clinical practice as a prerequisite before entry to APN education programs (N = 1767).

	Disagree n (%)	Neither Disagree nor Agree n (%)	Agree n (%)
Clinical nursing experience should be a prerequisite before entry to APN education programs (missing, *n* = 43)	77 (4.5)	31 (1.8)	1616 (93.7)
Knowledge, skills and general competence may be achieved through the specialization, and hence should not be a prerequisite (missing, *n* = 6)	1490 (84.6)	71 (4)	200 (11.4)
Clinical nursing experience from specialized wards should be a prerequisite before entry to APN education programs (missing, *n* = 17)	726 (41.5)	521 (29.8)	503 (28.7)
Clinical nursing experience should not be a prerequisite before entry to APN education programs (missing, *n* = 22)	1552 (88.9)	40 (2.3)	153 (8.8)
The educational institutions themselves should decide their own prerequisites (missing, *n* = 20)	1591 (91.1)	101 (5.8)	55 (3.1)
Prerequisites should be similar across educational institutions to ensure a national standard of APNs (missing, *n* = 19)	40 (2.3)	52 (2.9)	1656 (94.8)

APN = advanced practice nurse. Response alternatives Totally disagree (1) and Disagree (2) collated to ‘Disagree’. Response alternatives Agree (4) and Totally agree (5) collated to ‘Agree’.

## Data Availability

Data is available upon request to the author.

## Supplementary Materials

The following supporting information can be downloaded at: https://www.mdpi.com/article/10.3390/nursrep13030110/s1. File S1: The questionnaire.

## Public Involvement Statement

No public involvement in any aspect of this research.

## Guidelines and Standards Statement

This manuscript was drafted against the STROBE guidelines [24].
