# Supplementary material for: The Impact of Clinical Experience in Advanced Practice Nursing Education—A Cross-Sectional Study of Norwegian Advanced Practice Nurses’ Perspectives
Source: Nurs Rep. 2023 Sep 13;13(3):1304–17. doi: 10.3390/nursrep13030110 (PMC10538056; doi:10.3390/nursrep13030110)
Supplement: Supplementary file 1 [file nursrep-13-00110-s001.zip › nursrep-2562749-supplementary.pdf]

Supplement File S1. The questionnaire

(response alternatives in parenthesis)

What advanced nursing education do you have/are you studying? (advanced clinical generalist, cancer, critical care, emergency care, midwifery, nurse anesthetist, operating room, pedicatric, public health, other)

If you responded 'other', please specify (text)

Are you an advanced nurse or a student? (advanced nurse, student, neither)

What is your current position? (administration, clinician, leader, professional development, researcher, teacher, other)

If you are an advanced nurse: how many students in advanced nursing have you supervised throughout your career? (insert number)

Please report to what extent you disagree or agree on that clinical nursing experience impact on:

- **Development of an individual nursing identity**
- **Experience with communication with patients**
- **Experience with communication with relatives**
- **Experience with medical equipment**
- **Experience with basic procedures**
- **Experience with advanced procedures**
- **Experience with interprofessional collaboration**
- **Experience with documentation**
- **Experience with independent task solving**
- **Development of situational awareness**
- **Development of awareness of own role in teams**
- **Development of an ability to provide personcentred nursing**
- **Increased patient safety**
- **Increased efficacy**
- **Development of a feeling of security in the provision of nursing care**

(1=totally disagree, 2=disagree, 3=neither disagree nor agree, 4=agree, 5=totally agree)

If you have experience with supervising students in advanced nursing: Please report to what extent you disagree or agree on the following statements:

- **There is NO association between the number of years of clinical experience and nurses' ability to achieve the learning outcomes**
- **There is NO association between the nature of the clinical experience and nurses' ability to achieve the learning outcomes**
- **The students' ability to achieve the learning outcomes depends more on personal assets than on clinical experience**

(1=totally disagree, 2=disagree, 3=neither disagree nor agree, 4=agree, 5=totally agree)

Please report to what extent you disagree or agree on the following statements:

- **Clinical nursing experience should be a prerequisite before entry to advanced nursing programs**
- **Knowledge, skills and general competence may be achieved through the specialization, and hence should not be a prerequisite**
- **Clinical nursing experience from specialized wards should be a prerequisite before entry to advanced nursing programs**
- **Clinical nursing experience should NOT be a prerequisite before entry to advanced nursing programs**
- **The educational institutions themselves should decide their own prerequisites**
- **Prerequisites should be similar across educational institutions to ensure a national standard of advanced nurses**

(1=totally disagree, 2=disagree, 3=neither disagree nor agree, 4=agree, 5=totally agree)

Text responses:

What is the advantage of having clinical experience previous to entry to an advanced nursing program?

What is the disadvantage of having clinical experience previous to entry to an advanced nursing program?
